# A vaccination romance: Rider Haggard’s *Dr. Therne* (1898) in the vaccination debate

**DOI:** 10.1017/mdh.2023.1

**Published:** 2022-10

**Authors:** Juliana Broad

**Affiliations:** Independent Researcher

**Keywords:** H. Rider Haggard, Anti-vaccinationist, Smallpox, Compulsory vaccination, Conscientious objector, Nineteenth century

## Abstract

Henry Rider Haggard, the famed author of adventure romances, wrote the novel *Dr. Therne* (1898) in response to weakening compulsory smallpox vaccination laws, thus entering one of the most heated debates of the late nineteenth century. With *Dr. Therne,* Haggard aimed to intervene in the lives of the many working-class anti-vaccinationists who, from the 1850s onwards, mobilised to evade what they perceived as a gross – and targeted – extension of state power at the expense of individual rights. Recovering the novel, which has not yet received scholarly attention from historians of medicine, reveals the way fiction was called upon to change minds during a crucial period of Victorian medicine, one that witnessed a climactic shift in public health intervention. This article will examine the reception of *Dr. Therne* in various print media – middle-class London papers, medical journals and working-class, anti-vaccinationist publications – to consider some new dynamics of the debate which the disagreement over Haggard’s polemic exposes, including the perceived power of fiction (when properly priced and distributed) to change minds, and the contested role of the evangelical press. Additionally, a discussion of the different iterations of *Dr. Therne*, and a look at an exceptional anti-vaccinationist response in the form of a competing novel, illustrates that pro- and anti-vaccinationists alike contributed to a moment in late Victorian society when the role of fiction was considered a worthy contender in a debate ostensibly about fact.

‘*Absit omen!* May the prophecy be falsified!’^1^

In this foreboding call to arms, acclaimed Victorian novelist Henry Rider Haggard was not warning against the seduction of witches’ spells, Zulu divinations, or any of the other supernatural curiosities his readers would have come to expect from his globe-trotting adventure novels. Rather, his injunction comes at the beginning of *Dr. Therne*, a largely forgotten pro-vaccination propaganda novel that sought to illuminate the dangers of the burgeoning anti-vaccinationist movement in Britain. Haggard wrote his pro-vaccination morality tale at the end of the era of Victorian reformism, by which time its clear social purpose abutted a nascent scepticism towards professional medicine. The novel responded directly to weakening compulsory smallpox vaccination laws, entering the author into a controversy that had been engrossing doctors, lawmakers, intellectuals, laypeople and activists for over four decades. What can a novel by a preeminent imaginative writer tell us about the vaccination debate?

Using a variety of archival sources – including Rider Haggard’s correspondence with his editor, housed at Columbia University; articles and other materials collected by anti-vaccinationist Alfred Milnes, held by the London School of Hygiene and Tropical Medicine and newspapers from across England – this article details not only how Rider Haggard’s pro-vaccination polemic intervened in the vaccination debate, but also the ways in which the novel’s reception sheds light on some of the complexities of its participants. It begins by situating *Dr. Therne* in its historical context, emphasising the social unrest to which Haggard was responding. The article then examines the reception of the novel in various print media – middle-class London papers, medical journals and working-class, anti-vaccinationist publications – to consider what the fierce disagreement over Haggard’s intervention can tell us about the debate. This approach – making sense of *Dr. Therne* through its reception among various groups of readers – though well-established in histories of medicine, has never been used in reference to studying the vaccination debate. A discussion of the novel’s different iterations, and a look at an unusual anti-vaccinationist response in the form of a competing novel, shows that, far be it from an irrelevant minor novel, *Dr. Therne* was a lightning rod for public scrutiny. Whether pro- or anti-vaccinationists were able to successfully mobilise fiction to change opinion would be nearly impossible to prove; instead, this article demonstrates that *Dr. Therne* compelled virtually all parties of the reading public – whether or not they had a devout opinion about compulsory vaccination – to make a consideration until now unexamined: what place fiction had in a debate ostensibly about fact.

Since the mid-twentieth century, scholarship on compulsory vaccination in Britain has revealed the immense social, scientific and legal upheavals which both generated and responded to vaccination resistance. The debate captures many of the challenges faced by Victorian governments, which during this period were rapidly reinterpreting the legal frontiers of scientific expertise, state intervention and individual liberty – what public health historian Alison Bashford has summed up as the paradox of liberal governance.^2^ The many prominent intellectuals and ‘men of science’ who were vocal anti-vaccinationists – those who wrote publicly and provided the ‘oratorical invective’ that brought vaccination to Parliament’s attention^3^ – provide a fascinating entry point to the debate, one which is rarely overlooked in the many general interest works on the period.^4^ The early administrative development of the vaccination laws and alternative strategies for evading smallpox – particularly the vaccineless approach of compulsory quarantine and isolation put into full effect by the anti-vaccinationist town of Leicester – have also received attention, with historians generally concluding that anti-vaccinationists were able to effectively steward the ‘Leicester method’, as it is known, to reduce sickness and death.^5^ R.J. Lambert has even argued that, to the extent that the Victorian state took responsibility for citizens’ health, compulsory vaccination constituted an early precursor to the National Health Service.^6^

Starting in the late 1960s, historians began focusing on how anti-vaccinationists – often taking cues from abolitionists and anti-vivisectionists – mobilised to evade what they perceived as a gross extension of state power at the expense of individual rights. Roy MacLeod has discussed how changes in compulsory vaccination legislation effectively reinterpreted parental responsibility from the lens of criminal law, and established an influential distinction between treatment and prevention: preventing one’s child from receiving medical treatment amounted to wilful neglect, yet abstaining from *preventative* measures was a matter for parents to decide themselves.^7^ MacLeod has also examined how public opinion against vaccination played a role in shaping legislation, setting off a wave of new scholarship on what has come to be called the anti-vaccination movement.^8^ Scholars like Dorothy Porter, Roy Porter and Ann Beck disentangled the movement’s heterogeneous ideological undercurrents, which drew from diverse political, religious and scientific values, allowing it to sustain opposition to compulsory vaccination laws despite long-term developments such as more interventionist understandings of the state and the adoption of the germ theory of disease, which could have extinguished more uniformly motivated opposition.^9^

Nadja Durbach has, in recent years, both redrawn the map of the anti-vaccination debate and recalibrated our compass for navigating it. Durbach’s articles and book have illuminated the perspectives of working-class members of the movement, on behalf of whose conscience most prominent interlocutors in the vaccination debate deigned to speak. Uneven enforcement of the vaccination laws, which disproportionately penalised working-class people and women, was an important feature of the lived experiences of resisters and explains the composition of the movement more broadly, a dimension which has only recently come to light through her scholarship.^10^ Durbach has also done the most thorough accounting of the role of the gothic Victorian imagination in the debate, delineating in particular how the literary trope of the vampire was animated by anti-vaccinationists to represent blood-thirsty, aristocratic doctors, those who ‘literally and symbolically bled the people dry’.^11^ Durbach’s work makes clear how much the stakes have changed in understanding the vaccination debate, from disentangling the genealogies of state power to thoroughly de-romanticising the notion that anti-vaccination is a quaint, uncomplicated belief of the past.

Of course, histories of Rider Haggard have similarly changed tack. Hagiographies, biographies and bibliographies abound due to the lasting imprint Haggard has made on generations of British school curricula as well as readers’ psyches – so much so, in fact, that Haggard counted among his most eager readers both Sigmund Freud and Carl Jung. A new generation of Haggard scholarship, largely starting with the work of Wendy Katz, has focused on scrutinising the role and status of empire in Haggard’s work – and, indeed, the role Haggard played in empire, defending its theories of hierarchical difference among people and presenting a stage for the indulgence of heroic fantasies.^12^ Scholars like Norman Etherington have drawn similarities between Haggard and other imperial luminaries like Rudyard Kipling (who was also Haggard’s close friend), Joseph Conrad and Robert Louis Stevenson, although Etherington is more circumspect about the straight line running from empire into the heart of Haggard’s work.^13^ Nina Auerbach and Anne McClintock have articulated the cultural anxieties around women and empire, and make up only constellations in what is now a large body of scholarship that analyses the figure of the ‘Other’ in Haggard’s work.^14^ Recent work on Haggard has examined the essential role of illustration and serialisation in reading Haggard in the nineteenth century, with Kate Holterhoff creating an entire online digital archive of the hundreds of illustrations which accompanied Haggard’s serialised novels.^15^ Furthermore, although literary critics and scholars have probed Haggard’s writings in relation to psychoanalysis, anthropology and Darwinian evolution, those that touch on medicine have remained unplumbed.^16^

While *Dr. Therne* gets passing mention in most books and articles that attend to its author (one authoritative biography, reviewing Haggard’s oeuvre, refers to his forty-seven adventure novels, twelve novels of contemporary life and a singular ‘propagandist novel’^17^), the book is not the subject of any single monograph, chapter or scholarly article. In fact, the only work to take the novel as its somewhat sole subject is Richard Reeve’s short essay, which argues the novel’s indebtedness to Anthony Trollope’s *Doctor Thorne.* ^18^ Where *Dr. Therne is* discussed by scholars, it is usually taken on the terms of a larger thesis. For example, in her foundational study of Haggard, Katz’s treatment of *Dr. Therne* amounts to a paragraph, summed up in a single line: the book amounted to ‘yet another assault on Radicalism and its dishonesty’.^19^ Another study, on the spiritualist elements in Haggard’s writing, briefly discusses the novel’s indirect criticism of organised religion (a theme, it should be noted, which does not register in contemporary criticism of the novel).^20^

What, then, are we to make of this absence of *Dr. Therne?* Haggard wrote the novel as an explicit intervention into the lives of working-class anti-vaccinationists, entering himself into one of the most heated debates of the late nineteenth century, and yet the novel and its reception have been overlooked by historians of medicine and Haggard alike. Wendy Katz has argued that Haggard’s fiction – though not often described with the sobriquet ‘serious literature’ – was only ‘superficially innocuous’, with his ‘popular and accessible writing style’ making him an ‘imperial propagandist’ bar none.^21^ The following study of *Dr. Therne* – focusing on its context and critics – attempts a first understanding of how Haggard’s fiction was deployed to challenge hearts and minds during a crucial period of Victorian medicine.

## A novel with a purpose

In 1853, Britain became the first country to mandate that every citizen receive a smallpox vaccination. The Compulsory Vaccination Act was carried by the tide of interventionist legislation increasingly seen as necessary, as industrialisation continued apace, for the regulation of workplaces, unsanitary living conditions and poor relief. Yet, the law was an immediate flashpoint for resistance, since many Britons resented finding themselves targeted by the state for not just social, but also bodily, control. The 1853 act and its revisions, which included cumulative penalties for refusing vaccination, were controversial and poorly administered, a potent combination that allowed resisters to leverage their alliances with dissenting local officials to create widespread disruption and noncompliance. In Leicester, where anti-vaccinationists were elected to local office and thus had control over administering the law, one anti-vaccination rally of nearly one hundred thousand participants summarily decapitated an effigy of Edward Jenner, father of the smallpox vaccine.^22^

By the late 1880s, despite many changes to the law, resistance to compulsory vaccination had not abated, and Parliament appointed a Royal Commission to draft ways to improve its administration.^23^ After 7 years of deliberations, the commission issued a contentious final report with the main recommendation: allow conscientiously objecting parents to refuse to vaccinate their children. In 1898, an amended law passed with the conscientious objector clause, legally permitting objectors to refuse vaccination without – in theory – risk of fine or imprisonment. In practice, however, working-class Britons continued to be disproportionately targeted, as ‘who exactly was entitled to make a claim to possess a conscience, with its concomitant rights’ became the reconstituted barrier to legally abstaining from vaccination.^24^ Anti-vaccinationists circulated advice on how conscientious objectors could avoid unjust targeting, and maintained informal defence funds to help pay the fines of those denied exemption certificates.^25^

Henry Rider Haggard, a household name as the beloved author of adventure and romance novels, had witnessed the ravages of smallpox first-hand while travelling in Mexico, and saw the conscientious objector loophole as a regression in the interventionist legislation that had safeguarded British communities for decades. Anti-vaccinationists Haggard wrote, ‘think little of the disease because they have scarcely seen it at its dreadful work. What they lack is imagination’.^26^ If nothing else, Haggard had imagination, making his impetus for writing *Dr. Therne* typical of an era in which an earnest attempt to transform readers was seen as the first step in transforming the world – a position which made individuals both the agents and sites of social change.^27^ Indeed, some of the period’s most notable authors had used fiction to raise the alarm over the dangers of emerging medical sciences, especially vivisection: H.G. Wells’s *The Island of Doctor Moreau,* Wilkie Collins’s *Heart and Science* and Robert Louis Stevenson’s tale of Dr. Jekyll and Mr. Hyde were all published within roughly a decade of *Dr. Therne.* What Haggard earnestly hoped to accomplish with his novel-as-exhortation was not unreasonable.

His earnestness aside, Haggard’s involvement in the *vaccination* debate was unconventional – the typical media for social persuasion were lantern slides of smallpox victims, statistical reports and letters to newspaper editors, none of which were circulated by fiction authors – and so it comes as no surprise that adherents from both sides of the debate invoked his credentials as a novelist to either embolden or discredit him.^28^ While mainstream physicians quickly embraced *Dr. Therne* as enlightened, convincing anti-vaccinationists was another matter.

‘I sat up last night and read *Dr Therne.* It is dramatic all through, and though the subject is painful and unpleasant there is nothing in the treatment that strikes a jarring note’, wrote Haggard’s long-time publisher, Charles Longman. ‘The question of course is who will read it; you are of course going quite outside your regular clientele’.^29^ Longman had worked with Haggard on his canonical adventure novels – among them *She* and *King Solomon’s Mines* – and was grappling with Haggard’s rapid turn towards the political. As the *New York Times* noted, Haggard had drafted *Dr. Therne* in less than 4 weeks, starting immediately upon learning about the inclusion of the conscientious objector clause, and Longman tried to respond with equal urgency.^30^ (Part of Haggard’s gift was writing ‘at full speed, hardly stopping to think, so to speak’, as writer Henry Miller noted – Haggard’s best-known novel, *King Solomon’s Mines*, was written in a mere 6 weeks.^31^) In order to avoid a publishing conflict with Haggard’s forthcoming novel, *Swallow*, which was scheduled for spring 1899, Longman suggested that *Dr. Therne* immediately enter the market, forgoing a standard but logistically challenging serialisation, at the standard three shillings and six pence.^32^

The story pivots on James Therne, a young physician in the fictional village of Dunchester who, after being framed by a devious but distinguished rival doctor in town for criminal negligence in the death of a patient, is left in dire financial and professional straits. With little prospect of continuing his medical career – and on the verge of taking his own life – Therne agrees to the offer of a local benefactor, Stephen Strong, who promises to support Therne’s bid for Parliament – on the condition that Therne run as a Radical on an anti-vaccination platform. While Therne is opposed to anti-vaccinationists, he feels he has no choice but to accept the terms. Once elected, Therne helps usher in the conscientious objector clause, and, for 20 years, the people of Dunchester legally abstain from vaccination. When a smallpox-infected traveller comes into town, the epidemic spreads quickly, and, fearing for his own health, Therne vaccinates himself in the dead of night. His daughter Jane, who Therne never had vaccinated and who has contracted smallpox, walks in on him doing so. Before she succumbs to the disease, Jane tells her fiancé, Ernest Merchison, another doctor in town, of her father’s betrayal of the cause of anti-vaccination, in which she had firmly believed, and of her. Merchison outs Therne at his re-election meeting as an opportunistic fraud by forcing Therne to publicly bare his vaccination scar. Therne is ruined, and flees to the island of Madeira, leaving Dunchester to grapple with the deaths of over five thousand people.

Like the Victorian adventurers in colonial Africa upon whom Haggard based many of his stories, James Therne was a scientific man (with fatal personal ambitions) whom the reader could join on an extraordinary civilising mission. The reader breaks bread with Strong’s wife, a woman whose mind, when not engrossed in anti-vaccination literature, ‘occupied itself almost entirely with that well-known but most harmless of the crazes, the theory that we Anglo-Saxons are the progeny of the ten lost Tribes of Israel’.^33^ The perception of anti-vaccinationists as just one of several flavours of cranks – people ‘for whom life without indignation was impossible’ – was widespread at the time.^34^ As we will see, however, many readers were disappointed by Haggard’s departure from the exhilarating imperial romance featuring intoxicatingly racist depictions of Zulu kings to a rather quotidian countryside spectacle, where the exotic Other is found lace-capped on a sofa reading obscure pamphlets. Perhaps, by embedding his story among England’s ‘faddists’, Haggard was attempting to bring colonial ethnography home, infiltrating and revealing domestic oddities not so much to better understand the Other, but revealing the process by which the enlightened man – the medical man in particular – better understands himself.^35^ Yet trying to change the minds of those one depicts so deprecatingly appeared to be a losing strategy, one which Haggard’s editor sensed.

‘The A.V. people will abuse it’, Longman continued in his response to Haggard’s draft, referring to anti-vaccinationists, ‘but whether they will buy it largely I cannot say’.^36^ In public forums throughout the novel’s tumultuous reception, Haggard maintained that an author’s responsibility was to depict ‘disasters that may result in the future, as he sees those unborn events in the light of his own mind’, disasters which the anti-vaccinationists clearly did not fathom.^37^ In a highly partisan debate that was often staked out in equally partisan periodicals – with the anti-vaccinationists relying on their own journals and allied newspapers such as *The Star*, the *East London Observer* and the *Morning Leader* – Haggard was confident that fiction, in his hands, could appeal to the mind of the average Briton where scientific diatribes had failed. By considering *Dr. Therne*’s reception among three groups of readers – London’s middle-class periodicals, leading medical journals, and anti-vaccinationist literature – we cannot only evaluate whether Haggard succeeded, but also begin to uncover where and how these readers drew lines of demarcation between public health messaging and propaganda, science and mere speculation.

## ‘A work of pure imagination’: *Dr. Therne*’s readers

In its notice of forthcoming publications, *The Times* was the first to ask the question that remained a leitmotif throughout London’s middle-class press coverage: Why was Rider Haggard publishing a novel about, of all things, the conscientious objector? ‘A novel on the vaccination question seems a curious development even for modern fiction – and it is no less curious that the author should be Mr. Rider Haggard’.^38^

This sentiment was echoed across publications. *The Spectator –* a leading weekly during this period, which stressed ‘morality, good taste and wholesomeness’^39^ *–* located Haggard within the milieu of authors who had warped the novel into a tool of social reform: ‘There was a time when the scope of the novel was too exclusively confined to the full-dress functions. Nowadays we run to the other extreme, and degrade it to the level of a literary maid-of-all-work’.^40^ Whereas the adventure novel explored the weird and primitive from a safe distance, the realist novel, as critiqued by *The Spectator*, betakes itself ‘to the sphere of the abnormal, to the exploration of the charnel-house of humanity and to the study of mental, moral and even physical degeneracy’.^41^ Something particularly upsetting attended an investigation of one’s own backyard.

Haggard responded to this criticism directly, appealing to the virtue of realism in facilitating social improvement. Any person ‘who chances to have the welfare of childhood much at heart’ would be compelled to speak out against these dangerous developments, and that, furthermore, there was ‘nothing reprehensible in such a desire’. It was Haggard’s hope, ‘however futile’, that others will take up the cause and that ‘public opinion may bring about a change in the law’.^42^

While the *Manchester Guardian* picked up on the exchange to flippantly register appreciation that more novelists did not trouble themselves with social issues, the literary magazine *Academy*, catering to educated, middle- to upper-class readers, sombrely criticised Haggard’s nonstrategic attempt to educate working-class anti-vaccinators.^43^

If Mr. Haggard is really intent on proselytising he will direct that it be issued in tract form at a popular price as well as in its present three-and-sixpenny form. The ‘conscientious objectors’ who now come before the magistrates day after day and waste the time of the Court are not to be reached by three-and-sixpenny pamphlets, but – if at all – by penny ones.^44^

The *Athenaeum*, largely considered the leading journal of literary criticism of the late Victorian period and indispensable to those in both literary and scientific circles, found *Dr. Therne* little more than ‘a popular means of advertising the lurid ways of a fell disease’.^45^ *The Times* gave their brief review a few days later, confirming their intuition of the novel’s incongruity: *Dr. Therne* was ‘scarcely calculated to increase the literary reputation of its distinguished author’.^46^

*Dr. Therne*’s tepid reception was due, in part, to what the conservative *Morning Post* suggested was its underlying ‘common sense’.^47^ Apart from the first two chapters set in Mexico, which introduced readers to the horrors of smallpox, *Dr. Therne* had neither the adrenaline of adventure nor the fascination of the exotic; for many of these readers, *Dr. Therne* simply confirmed long-established prejudices.

Although it was started as an outlet for power-homing critiques, by the late nineteenth century, the London satirical weekly *Punch* had largely shaken off its radical undertones in exchange for light social commentary.^48^ Its target audience was middle-class, yet its readers were legion, making *Punch* one of the most widely read periodicals of the era. Famous for its bracing satire and political cartoons, as well as its bevy of influential writers and artists, the magazine often featured short parodies, unauthorised sequels and general pastiches of culture both high and low.

In early February 1899, under the banner of ‘Mr. Punch’s Dramatized Novels’, there appeared a two-column, three-act reinterpretation of *Dr. Therne.* ^49^ Providing a glimpse of the climate of suspicion which was to characterise the coming *Doctor’s Dilemma-*era Edwardian period, *Punch*’s re-telling of *Dr. Therne* painted *both* physicians – Therne and Merchison – as conniving, selfish and not to be trusted. Not only does Therne, the anti-vaccinationist doctor, vaccinate himself in the dead of night, but also Merchison – the presumptively upstanding fiancé of Therne’s daughter – admits that, although he promotes vaccination for his patients, he would never get the procedure done on himself. In the parody’s final scene, Therne makes Merchison bare his arm, which lacks the token vaccination scar:

*Merchison (dismally).* You’ve found me out.

*Therne (genially).* And you’ve found *me* out, eh? Well, let’s strike a bargain. You say nothing about me and I’ll say nothing about you. You agree?

*Merchison.* I agree.

*Therne:* Very well. Then Donkeyster keeps its Member [of Parliament]. And, by the way, under these circumstances I’ve no objection to your marrying my daughter. We scoundrels ought to have a fellow feeling for each other. *(Shaking him by the hand.) Good* morning.

(Curtain.)

This conspiratorial back-slapping epitomises what George Bernard Shaw soon thereafter characterised as doctors’ ‘conspiracy against the laity’, which was worse than that of other professional conspiracies only because the medical profession was ‘less suspected’.^50^

The only outlier to this general middle-class scepticism of Haggard’s proselytising was *The Outlook*, a weekly conservative magazine that often sounded the trumpet for imperial affairs.^51^ *The Outlook* was confident that *Dr. Therne* could ‘appeal to the emotions of the uninstructed and to the intellects of those who are better informed’ and ‘prevent the development of many objectionable consciences’.^52^ While the stewards of late-Victorian literary sensibility had reached consensus on Haggard’s misguided foray into medico-social realism, England’s medical professionals lost no time in comparing him to Zola and Ibsen.^53^

Under the title ‘A Vaccination Romance’, the *British Medical Journal*, a leading platform for pro-vaccination physicians, applauded *Dr. Therne*’s strong didactic message and the ‘unsparing realism’ with which it explored Therne’s contradictions.^54^ The *BMJ* hoped that by presenting a dystopian, virus-ravaged future, the novel could spark a renewed sense of urgency in otherwise complacent individuals ‘who would never trouble their heads further than to be revaccinated when an epidemic is threatening’, and remind readers of how quickly charlatans could trick communities into losing their hard-won immunity.^55^

Both the *BMJ* and *The Lancet* underlined the novel’s relevance by referring to recent episodes, warning readers how urgent, still, was their battle against ‘the honest fanatic, the designing agitator and the ignorant dupe’, each of which appears in the novel and in the debate over vaccination.^56^ The *BMJ* maintained that this tale was no mere fiction: ‘The vaccination of the anti-vaccinationist leader in fear of the epidemic is an incident not without historical basis in fact’.^57^ A few weeks later, on 17 December, *The Lancet* re-iterated that the novel was sensational for exactly how closely it mirrored reality: the final scene at the town meeting ‘was probably prompted by an occurrence which recently took place in London in connexion with the adventures of a gentleman whose career would have had the deepest interest for Mr. Haggard a few years ago’.^58^

Although the exact events the *BMJ* and *The Lancet* were alluding to remain unknown, a strikingly similar episode (from outside of London though it was) would suggest that stories of fraudulent anti-vaccinationist leaders were not entirely unheard of. In 1895/6, just 2 years before *Dr. Therne* was published, a smallpox outbreak ravaged the anti-vaccinationist stronghold of Gloucester. As the epidemic loomed, Walter R. Hadwen, one of the leading anti-vaccinationist physicians and voice of the movement, was rumoured to have had himself and his children surreptitiously vaccinated.^59^ Whether Haggard was aware of this episode, or took it as his inspiration, is unclear; regardless, the apparent reincarnation of the ignoble events of Gloucester in the form of Haggard’s novel prompted eager counter-narratives, as will be discussed later.

Despite the conflicting accounts of what happened in Gloucester, *Dr. Therne*’s apparently plausible story excited the major organs of the medical profession: the book illustrated in striking detail the chicanery of anti-vaccinationists against which doctors had so long warned. Not even Haggard’s portrayal of multiple conniving, self-serving physicians seems to have discouraged the medical community. After all, the doctor of the late-Victorian novel was not uncommonly a character whose growing scientific drive to knowledge was at odds with social compassion.^60^ Yet, the medical press’s reviews of *Dr. Therne* featured no hand-wringing; rather, a few unsavoury doctors seemed a small price to pay for the bounty of the pro-vaccination moral. As the *BMJ* had identified in connection with the anti-vaccination debate only a year earlier, the written word itself could act as a type of inoculation: what mattered was that it got the job done.^61^

*The Lancet* promised that while vaccination ‘does not sound a tempting theme for those who seek oblivion in the perusal of stories’, fewer scenes in all of fiction had been better told, an acknowledgement meant to reassure readers of *Dr. Therne*’s durability in a debate that often featured more histrionics than scientific rigour.^62^ Literature had finally ‘begun to mention us with approval and even champion our causes’ reported *Medical News*, a Philadelphia weekly, and *Dr. Therne* was sure to be a strong ally ‘ – if the “antis” ever read anything except their own lying pamphlets’.^63^ Not only did anti-vaccinationists read *Dr. Therne*, but, like the medical community, they also saw the novel as a vindication of their own staunch opinions.

The front page of *The Star*, a London-based working-class daily and reliable critic of compulsory vaccination, often exposed what it claimed were the devious operations of pro-vaccinators.^64^ *Dr. Therne*, as an intensely dogmatic tract penned by a famous writer, was a symptom of such unchecked power, and *The Star* devoted a full column to its review.

Mr. Rider Haggard has turned from the production of witch’s heads, hot pots, misty people, and other such-like sober actualities, to try his hand at last on a work of pure imagination. The advantages of being vaccinated are now his theme; and there at last he soars to that astral plane whereon fancy roams emancipated from every slightest trammel of mere terrestrial fact.^65^

Anti-vaccinationists were especially attentive to Haggard’s factual missteps. For example, *The Star* pilloried Haggard for mixing up the titles of a vaccine-administering doctor and an administrative official: ‘The author of a work of pure imagination cannot be supposed to know the difference between a public vaccinator and a vaccination officer, and Mr. Haggard does not know it’.^66^ But whether these faults were the products of apathy or incompetence seemed to matter little. *The Star* considered *Dr. Therne* to be the kind of heedless interjection masquerading fiction as fact which was responsible for mucking up confusion over vaccination in the first place. Likening vaccination to a fairy tale, *The Star* underscored the potency of both: neither were ‘real’, but both had ‘a very real existence’, a warping influence on the imagination that, in the case of vaccination, could foster reckless belief in an unscientific practice, and thus endanger one’s health.^67^

*The Star* further condemned what they saw as an outright injustice in the comparison of the strategies used in England by anti-vaccinationists to those used in Africa. Haggard, *The Star* accused, writes how ‘“the terrible rule of isolation known as the improved Leicester system” is virtually “the same plan” as that of African savages who surround with guards the kraal in which smallpox has broken out’. This was, *The Star* railed, a ‘slanderous falsehood’.^68^ Haggard’s comparison of the Leicester method of containment with strategies deployed by ‘the natives of Africa’ (which is the language Haggard actually used) – and the anxious rebuttal it provoked among the anti-vaccinationist press – adds to the growing picture of the contempt with which English anti-vaccinationists regarded their peripheral counterparts.

The *Vaccination Inquirer –* a journal founded in Manchester in 1879 by one of the movement’s leaders, William Tebb, which was the voice of the anti-vaccination movement^69^ *–* took a less hostile approach than *The Star*, performing mock disappointment at what was, nevertheless, an aggressive and unusually accessible reproach of their side:

The persons who, in respect of this book, are most to be pitied are those who are misled by Mr. Haggard’s reputation as a story-teller into buying *Dr. Therne* in the expectation of a few hours of pleasant amusement. Save when it is enlivened by some really brilliant flashes of ignorance, the book is wearisome to a degree beyond words.^70^

Whereas middle- and upper-class readers shuddered at Haggard’s unexpected attention to local oddities, anti-vaccinationists chastised Haggard for what they saw as his complete departure from reality. The enthusiastic endorsement from medical professionals, including the Jenner Society, met by the dissatisfaction of nearly everyone else, from the literary to working-class worlds, would seem to indicate that *Dr. Therne* was not the headline-grabbing success that Haggard saw with titles such as *King Solomon’s Mines.* It had clearly tested the limits of what a novel – even in the name of public good – should be made to do. By February 1899, Longman told Haggard: ‘We have sold about 4400 of the 3/6 edition of Dr. Therne – it does not sell very fast now, but it does keep moving: 100 have gone in the last 10 days. We printed 10,000, so that we have over 5000 left, and I fear that if a 6d edition were now published nearly all this stock would be rendered worthless’.^71^ Nevertheless, *Dr. Therne* reappeared as both a sixpenny edition and, before that, as a lavishly illustrated, widely circulated serial from a parish press.

## *Dr. Therne*: Evangelist

In 1900, 2 years before the reappearance of *Dr. Therne* as a sixpenny edition, the Society for Promoting Christian Knowledge (SPCK) serialised the novel in their parish magazine, *The Dawn of Day.* The ‘interesting tale’ was ‘well suited to the requirements of a parish magazine’, noted the *Oxford Times.* ^72^ Serialisation was the vanguard publishing form of the Victorian era, although the publication of *Dr. Therne* in instalments was not entirely routine, since the novel was serialised a year and a half after its initial publication. As we saw above, Haggard’s publisher Charles Longman saw the drawn-out process of serialisation as a losing strategy in the quicksand of public opinion. As much recent scholarship has suggested, however, by the mid-nineteenth century, the initial economical value of serialisation had been partly eclipsed by its triumph as a cultural form uniquely suitable to the age.^73^

*The Dawn of Day*, founded in 1878, was a parish magazine that counted among its contributors the poet Christina Rossetti.^74^ Though originally started as a magazine for Sunday school children, by 1885, the SPCK was determined to reach more adults so that it would be ‘more suitable to enlighten the mass of the people upon the position and claims of the Church of England’.^75^ This period was marked by a surge of evangelical groups forming tract societies not only to reach ever-larger swaths of believers, but also to think through how new scientific discoveries related to their personal faith and church teachings.^76^ Estimates of *The Dawn of Day*’s circulation vary. The society’s own contemporary history stated that the parish magazine started with a circulation of only a few thousand, but by 1898 (the time of the chronicle’s publication) had reached ‘considerably over half a million, which increases year by year’.^77^ Another contemporary estimate put the monthly circulation at 750 000.^78^ Even if the true circulation was half these estimates, the publication’s reach would be impressive: the most wide-reaching periodicals of the day, such as *Tit-Bits* and *Pearson’s Weekly*, had circulations of roughly half a million.^79^

Notices for the parish magazine featuring *Dr. Therne* appeared throughout the country, in the *Oxford Times*, Exeter’s *Western Time*s and North England’s *Wakefield and West Riding Herald* – the magazine, the latter wrote, contained ‘the second chapter of Mr Rider Haggard’s fascinating story “Doctor Therne”’.^80^ Each instalment in *The Dawn of Day*’s 12-month run featured three pen-and-ink sketches by the water-colourist W.S. Stacey, amounting to thirty-six illustrations of the novel’s most evocative scenes.

The few reader responses to the serialisation captured in the press largely hit upon the same note: church doctrine would do well to keep away from fiction. In an unsigned letter to the editor of the *East London Advertiser*, one concerned reader laid out his credentials for balanced critique. He was a member of the English Church Union, a reader of Rider Haggard (who was ‘clearly a man of genius’) and an avowed believer in the science of vaccination. Thus, he could justify his claim: ‘I am not an anti-vaccinator’, he wrote, ‘But I hate trickery, especially pious trickery’. Why, he asked, ‘publish a severely controversial, one-sided story in a purely (adapted so at least) evangelical, moral and religious print, no doubt “taken in” by thousands of persons who sincerely believe in the curse of vaccination’?^81^

Another concerned reader wrote his thoughts in *Truth* magazine, arguing that a parish magazine should flirt with neither science nor fiction. ‘It is not surprising’, he wrote:

that in all parts of the country the subscribers of parish magazines who suddenly find Mr. Rider Haggard’s polemical romance foisted upon them are highly indignant, and many who do not differ from Mr. Haggard’s personal views recognise that this method of disseminating disputable opinions is illegitimate.^82^

Not only, this author claimed, were there plenty of church members who would likely disagree with Haggard’s position on the subject, but even those who might actually agree would not approve of this method of persuasion. Additionally, the author notes how the church was already prey to schismatic differences of opinion ‘without the ecclesiastical authorities going out of their way to multiply them by identifying themselves with non-religious controversies’.^83^ If the clergy really wanted to advocate for compulsory vaccination ‘as an article of faith’, then, this author suggested, ‘they should at least rely upon argument, not upon fiction. There is no more foolish controversial weapon than the type of novel with a purpose which Mr. Haggard is producing’.^84^

The *Church Sunday-School Magazine* had, a few years prior to this episode, maintained that ‘all babies must be registered, vaccinated and christened before they could be considered English Christian children’.^85^ Many anti-vaccinationists drew parallels between medical liberty and religious liberty, a claim that was particularly salient given what many considered to be the ‘very real relationship’ between established religion and vaccination.^86^ Yet, vaccination (and, earlier, inoculation) also posed an existential threat to the cosmology that braided bodily with spiritual purity, a dynamic that was especially true with evangelicals, who highlighted the ‘fragility of the child’ as a symbol of its purity and grace. Although most discussions of the anti-vaccination movement acknowledge its religious and spiritual undercurrents, the relationship between popular evangelical writing and vaccination – as this brief look at *Dr. Therne’*s serialisation in the evangelical press demonstrates – warrants further exploration.

## The return, and end, of *Dr. Therne*

The novel’s second edition – *Dr. Therne: Anti-Vaccinist*, published in March 1902 by London’s George Newnes – appears to have been reissued in indirect response to the fallout from the 1898 conscientious objector clause. There was a renewed sense that working-class Britons were again being targeted: critics pointed out that the process of receiving an exemption entailed overcoming significant bureaucratic and financial barriers, which effectively kept the working class in states of noncompliance.^87^ For example, at an anti-vaccinationist conference in Lincoln, ‘it was remarked that persons at present had to lose half a day’s time pay for the production of the birth certificate and the exemption certificate and got insulted into [*sic*] the bargain’.^88^

In May, *The Lancet* encouraged its readers to recall that novel with purpose, *Dr. Therne.* The second edition had just been released, and the author’s new introduction explained why. As Haggard wrote, he was releasing the second edition of the novel after having received an unusual letter from a reader. ‘As our pages are read by an audience already in no doubt as to the benefit of vaccination’, the article opined, *The Lancet* was unaccustomed to featuring stories of successful conversions from disbelief, but now made an exception.^89^

After having read *Dr. Therne*, George J. Battle, a resident of Rushden, a small village with its own Anti-Vaccination League chapter, had been compelled to abandon his decades-long belief in ‘theories concerning vaccination’.^90^ Haggard included this convert’s story in the new introduction to demonstrate that the novel’s persuasive power seemed to surpass that of the expert testimony and statistical figures so often appealed to by advocates of vaccination. *The Lancet* was eager to share the words of a convert:

In 1898 I was fined for non-compliance with the Vaccination Act and I have since obtained exemption certificates for my three children under the conscientious objector clause in the Act of 1898. Your ‘Dr. Therne’ has made me think that non-compliance with the Vaccination Act is the worst course to take and I shall not only have my children vaccinated but shall submit to the operation myself.^91^

The second edition was prompted by Haggard’s worry that *Dr. Therne*, though well-meaning, was stymied by its inability to reach the right audience. Haggard was moved by readers like George Battle, as well as concerned physicians, like one doctor who wrote, pleading for a cheaper edition of the novel that he could share with anti-vaccinationists: ‘Will you not lift your voice on the side, not merely of right, but of what is almost vital to the people? Could you not bring out this book in a cheap form’?^92^

Despite Longman’s original apprehension, by 1902, it therefore seemed that a sixpenny edition would do more good than harm. Longman obliged by licencing the book to George Newnes, founder of the mass-circulation weekly magazine *Tit-Bits.* Newnes had experience appealing to a wide audience and printed a sizable 200 000 copies of the second edition.^93^ The ‘cheap reprint’ as the *Times Literary Supplement* presented it, was now, as promised, ‘made available for the million on the strong advice of a medical expert’.^94^ Inside the book, after the title page, and sharing a spread with the author’s introduction, was a printed advertisement for bile beans – for the treatment of biliousness – and a coupon for readers of *Dr. Therne.*

Although there is little evidence of the second edition’s impact in print media besides scant advertisements, I would suggest that this was not the goal. The multiple iterations of *Dr. Therne* can be understood as one prong in a full-fledged campaign to drown out anti-vaccinationists using every tool possible. The second edition was also issued to meet a different challenge. Although publicly framed as a generous response to the demands of concerned readers, the sixpenny edition must also be recognised as a targeted rebuttal of a surprising piece of counterpropaganda authored by a leading anti-vaccinationist.

On 2 December 1901, the *Vaccination Inquirer* ran a small announcement for a forthcoming novel by the National Anti-Vaccination League’s (NAVL) president. The journal intended its readers to purchase the new book, available at the League’s offices in London, before the *Vaccination Inquirer* ran a more substantial review: ‘We are reserving our more detailed notice till the next issue of the *Inquirer*, when we hope many of our readers will be able to follow our appreciation of this happy idea of our President’s with a greater interest because of their own knowledge of the book’.^95^

The book was *Lord Dunchester, or the End of Dr. Therne: An Autobiography.* This unlicenced sequel to Haggard’s *Dr. Therne*, written by anti-vaccination league president Lieutenant-General Arthur Phelps, was not printed and distributed by the League, but published by London’s Swan Sonnenschein and Co. The publishers were accustomed to working with authors critical of vaccination, especially when it came to the lack of statistical support for vaccination’s efficacy – an oft-distorted method of persuasion both criticised and weaponised by Phelps in his response to Haggard.^96^

*Lord Dunchester* opens with Dr. Therne pondering the ruins of his life from exile in Madeira, where he realises that, despite his eleventh-hour vaccination, he has fallen ill. After contacting the London pharmacy that supplied the calf lymph to inquire about its purity, the pharmacy threatens to take legal action should he publish any slanderous reports against them. Step-by-step, Therne’s confidence in the efficacy – and ethics – of vaccination unravels. Despite his growing suspicions, and in a last-ditch effort to rebuild his reputation, Therne returns to Dunchester in order to fund a new hospital wing devoted to vaccination. There, Therne learns that Dr. Graunsh, Dunchester’s public vaccinator, had fabricated the morbidity statistics from the smallpox epidemic that closed *Dr. Therne* and ended the eponymous character’s career. Wyley, Therne’s trustworthy steward throughout the sequel, puts it thus: ‘I believe there was some exaggeration about those figures. You know how enthusiastic Dr. Graunsh is’.^97^

Statistics, just like the realist novel, had the power to elucidate complex social problems by positing that a universal could be made out of a particular.^98^ Throughout the nineteenth century, social reform movements used statistics to justify claims to scientific knowledge, and undergird the authority to institute interventionist policies, a trend that was maintained throughout the compulsory vaccination debate. Pro- and anti-vaccinationists often used the same statistics – whether birth and death rates or morbidity and vaccination figures – to make contradictory claims about vaccination’s safety and efficacy, not uncommonly leading to widespread confusion exacerbated by the proliferation of countervailing print media.^99^ ‘This state of affairs satisfies no intelligent citizen’, commented George Bernard Shaw in 1901, in the midst of a smallpox outbreak.^100^ Shaw held that until professional statisticians – and not, he specified, medical professionals – could confirm the value of statistics to medical science, the public would continue to question which side had it right. ‘For my own part – and I believe I represent in this matter almost every educated person under 40 in London who has ever given any thought to the question – I no longer believe that the vaccinist case has been either proved or disproved’.^101^

Phelps packed *Lord Dunchester* with references to these statistical debates, signposting for attentive readers the relevant reports and medical research that disproved vaccination’s efficacy. In one passage, Therne reflects, ‘I did not then know that calf lymph was capable, as shown by Mr. Jonathan Hutchinson’s researches, of producing symptoms indistinguishable by experienced surgeons from those of the horrible disease from which I had been suffering’.^102^ Phelps assumed of his audience either a high degree of familiarity with the debates – thereby providing them a way to reaffirm their beliefs – or a near-academic eagerness to learn more about the true nature of vaccination.

The anti-vaccination press had criticised *Dr. Therne* for its undisciplined descent into ‘pure imagination’, and hoped that Haggard would ‘make a special study’ of *Lord Dunchester*: ‘He will find that there is more truth in General Phelps’s fiction than in his own, and that the names of anti-vaccinist authors and of their works are genuine, and will guide him, if he chooses, to the sources of the truth on our question’.^103^

If *Lord Dunchester* was released a mere 2 years after *Dr. Therne*, giving readers scarcely any time to read the original to which Phelps was responding, why did he bother? There is reason – backed though it is by scant evidence – to believe that Phelps was motivated by personal animus as much as political commitment. During the aforementioned epidemic of 1895/6, Walter R. Hadwen – a leading anti-vaccinationist physician in Gloucester – was rumoured to have had himself and his children surreptitiously vaccinated as the epidemic loomed. Yet, as Hadwen’s biographers contest, it was not Hadwen who betrayed the cause, but ‘another notable anti-vaccinationist of Gloucester, a newspaper proprietor’ who actually did the ‘violence to his staunchly held principles by accepting vaccination’.^104^ Contemporaries, like Gloucester’s Medical Officer of Health, and some later historians have suggested that the man in question was none other than Arthur Phelps, who owned the *Citizen.* ^105^ Speculation around whether Phelps saw a familiar story – rumoured or true, personal or political – reflected at him in *Dr. Therne*, and whether this personal indignity prompted his response, certainly adds a layer of intrigue.

However, given what we know of Phelps, another explanation may be more likely. Though initially optimistic about the development of the conscientious objector clause, Phelps himself eventually lost faith, sensing that it operated as a release valve that deflated, rather than energised, the movement. A year before *The End of Dr. Therne* was published, he wrote as much to his sister:

The concession of a conscience clause has acted as a narcotic to many, who are content to save their own skins, and leave others to fight for the cause. Our subscriptions have fallen off, and at the Annual Meeting in a fortnight we shall probably make rather a poor show.^106^

Phelps was the leader of a movement struggling to maintain its direction and urgency after a long-sought, yet somewhat compromised, victory. After all, most committed anti-vaccinationists felt that the adoption of the conscience clause was one step on the long road to complete abolition of compulsory vaccination. To witness its disappointing results – parents keeping their heads down, getting their exemption certificates and withdrawing the movement’s financial base – may well have driven Phelps to the unconventional results this article has discussed.

Despite advertisements placed in many of the same papers as *Dr. Therne* – the *Saturday Review*, the *Speaker*, *Athenaeum* and the *Academy* – *Lord Dunchester* provoked almost no response outside of the *Vaccination Inquirer.* As authors, Haggard and Phelps could not have been more different. For Haggard, *Dr. Therne*, in content and reception, was a career aberration; Phelps, on the other hand, wrote from the crucible of the vaccination debate, spending the latter part of his life ceaselessly defending anti-vaccination as president of the NAVL, and often coming under fierce personal attack.^107^ Haggard earnestly dedicated *Dr. Therne* to the Jenner Society, and Phelps, tongue-in-cheek, dedicated *Lord Dunchester* to ‘the sincerity, born of ignorance, of the members of all societies which mind other people’s business’.^108^ Both authors were compelled to take the debate into the realm of fiction, where both, largely, failed to garner the decisive, debate-ending support they sought.

## Conclusion

Whether or not their polemics changed one mind or thousands, Haggard and Phelps both constituted late-Victorian ‘merchants of doubt’ in their attempts to construct and propagate competing world views of medical authority, state intervention and individual responsibility around the vaccination debate. In considering the reception of *Dr. Therne* in England, this article has demonstrated how different groups of readers generally interpreted the book in accordance with their previously held positions in the debate. The London middle-class press was bemused, conceding the importance of the cause but rejecting its presentation in a novel. Physicians, exhibiting enthusiastic support, saw the beneficent power of vaccination reflected back at them, and championed *Dr. Therne* as the newest weapon in their armoury for converting anti-vaccinationists. The anti-vaccinationist press feigned amusement, identifying *Dr. Therne* as the excrescence of an overzealous novelist.

Yet, that does not mean that the novel had no impact. Clearly, there was a perceived need for a strong rebuttal which could challenge Haggard in style, substance and scope. The responses examined in this paper suggest that while *Dr. Therne* may have been, put bluntly, ‘rather poorly received’,^109^ a study of its various iterations and responses illuminates some new dynamics within the anti-vaccination debate. Among these are the perceived power, yet clear limitations, of fiction to play the galvanising role among readers that was anticipated by its authors; the scorn with which British anti-vaccinationists viewed being associated with – or worse, outright compared to – the fictional Africa of imperial romance stories; and the tension and conflicts present in the mobilisation of the parish press in favour of pro-vaccination propaganda. Unearthing the reception of *Dr. Therne* in the United States and continental Europe – where editions were released by Longmans and Tauchnitz, respectively – may portent interesting points of comparison, as would examinations of later British editions.

When it comes to understanding anti-vaccination in this period, long abandoned is the idea that a belief in science was the only factor shaping one’s attitude – although, as Phelps saw it, dubbing something ‘scientific’ could ‘gild any absurdity so as to make the British public swallow it’.^110^ Rather, an amalgam of political ideologies and social forces shaped peoples’ outlook and actions. In addition to establishing the study of *Dr. Therne* as a unique dimension of the vaccination debate, this article proffers the study of fiction more broadly as a way to trace these complex contours of belief. Although, as authors, Haggard and Phelps were undoubtedly at odds about the merits of vaccination, what emerges from an examination of their books is rather less clear-cut than the epithets ‘pro-’ and ‘anti-vaccinationist’ would suggest. Readers who might not hesitate to get their children vaccinated *did* pause at the use of fiction to compel others to do so. A follower of the Church of England might not have batted an eye at compulsory vaccination, but found deep disturbance in the Church promulgating such a view. As exhuming *Dr. Therne* shows, these messy, complicated beliefs may reveal themselves best not when sharpened by fact, but when diffracted through fiction.

## Footnotes

1

H. Rider Haggard , Dr. Therne (London: Longmans, Green, and Co., 1898), viii.

2

Alison Bashford , Imperial Hygiene: A Critical History of Colonialism, Nationalism and Public Health (London: Palgrave Macmillan, 2004), 40 .

3 Roy MacLeod, ‘Law, Medicine and Public Opinion: The Resistance to Compulsory Health Legislation 1870–1907’, *Public Law* (Parts I and II, Summer 1967), 107–28 and 189–211. Among these men are the co-discoverer of the theory of evolution by natural selection, Alfred Russel Wallace; Herbert Spencer; physician Charles Creighton and microbiologist Edgar Crookshank.

4 For example, Stuart Blume , Immunization: How Vaccines Became Controversial (London: Reaktion Books, 2017), 134 .

5 Dale L. Ross, ‘Leicester and the Anti-Vaccination Movement 1853–1889’, *Leicestershire Archaeological and Historical Society*, 43 (1967–8), 35–44; Stuart M.F. Fraser, ‘Leicester and Smallpox: The Leicester Method’, *Medical History*, 24, 3 (1980), 315–32. Despite being published by the National Anti-Vaccination League, J.T. Bigg’s book remains the definitive source on Leicester’s anti-vaccinationist activities: J.T. Biggs , Leicester: Sanitation versus Vaccination (London: National Anti-Vaccination League, 1912).

6

R.J. Lambert , ‘A Victorian National Health Service: State Vaccination 1855–71’, Historical Journal, 5, 1 (1962), 1–18 .

7

Roy MacLeod , ‘Medico-Legal Issues in Victorian Medical Care’, Medical History, 10, 1 (1966), 44–9532402510.1017/s0025727300010632PMC1033555.

8 MacLeod, *op. cit.* (note 3), 114.

9

Dorothy Porter and Roy Porter , ‘The Politics of Prevention: Anti-Vaccinationism and Public Health in Nineteenth-Century England’, Medical History, 32, 3 (1988), 231–52306390310.1017/s0025727300048225PMC1139881; Ann Beck , ‘Issues in the Anti-Vaccination Movement in England’, Medical History, 4, 4 (1960), 310–211368836510.1017/s0025727300025643PMC1034558.

10

Nadja Durbach , Bodily Matters: The Anti-Vaccination Movement in England, 1853–1907 (Durham: Duke University Press, 2005).

11
*Ibid.*, 142.

12

Wendy Katz , Rider Haggard and the Fiction of Empire (Cambridge: Cambridge University Press, 1987).

13

Norman Etherington , Imperium of the Soul: The Political and Aesthetic Imagination of Edwardian Imperialists (Manchester: Manchester University Press, 2017). See his earlier ‘Rider Haggard, Imperialism, and the Layered Personality’, *Victorian Studies*, 22, 1 (1978), 71–87.

14

Nina Auerbach , Woman and the Demon: The Life of a Victorian Myth (Cambridge: Harvard University Press, 1984); Anne McClintock , Imperial Leather: Race, Gender and Sexuality in the Colonial Context (New York: Routledge, 1994).

15

Pascal Fischer , ‘The *Graphic* She: Text and Image in Rider Haggard’s Imperial Romance’, Anglia: Zeitschrift für Englische Philologie, 125 (2007), 266–87; Julia Reid, ‘“Gladstone Bags, Shooting Boots, and Bryant & May’s Matches”: Empire, Commerce, and the Imperial Romance in the *Graphic*’s Serialization of H. Rider Haggard’s *She*’, *Studies in the Novel*, 43, 2 (2011), 152–78. See the *Visual Haggard* archive at https://www.visualhaggard.org.

16 For representative examples of these works, see Meegan Kennedy , Revising the Clinic: Vision and Representation in Victorian Medical Narrative and the Novel (Columbus: Ohio State University Press, 2010); Gerald Monsman , ‘Of Diamonds and Deities: Social Anthropology in H. Rider Haggard’s *King Solomon’s Mines*
’, English Literature in Transition, 43, 3 (2000), 280–97; Kate Holterhoff, ‘Egyptology and Darwinian Evolution in Conan Doyle and H. Rider Haggard: The Scientific Imagination’, *English Literature in Transition*, 60, 3 (2017), 314–40.

17

Morton Cohen , Rider Haggard: His Life and Work, 2nd edn (Hutchinson: Palgrave MacMillan, 1968), 219 .

18

Richard Reeve , ‘Henry Rider Haggard’s Debt to Anthony Trollope: *Dr Therne* and *Dr Thorne*
’, Notes and Queries, 63, 2 (2016), 274–8. See also Reeve’s book, *The Sexual Imperative in the Novels of Sir Henry Rider Haggard* (London: Anthem Press, 2018), which considers *Dr. Therne* an exception to his main thesis.

19 Katz, *op. cit.* (note 12), 54.

20 John Senior, ‘Spirituality in the Fiction of Henry Rider Haggard (unpublished PhD thesis: Rhodes University, 2003).

21 Katz, *op. cit.* (note 12), 154.

22 Nadja Durbach, ‘“They Might as Well Brand Us”: Working-Class Resistance to Compulsory Vaccination in Victorian England’, *Social History of Medicine*, 13, 1 (2000), 45–62: 57.

23 For one helpful comparison of how compulsory vaccination played out in the British colonies during this period, see Sanjoy Bhattacharya , Mark Harrison and Michael Worboys , Fractures States: Smallpox, Public Health and Vaccination Policy in British India 1800–1947 (Hyderabad: Orient Longman, 2005).

24 Durbach, *op. cit.* (note 10), 172.

25 ‘Our Advice to the Conscientious Objector’, *The Star*, 22 September 1898; Durbach, *op. cit.* (note 10), 101.

26 Rider Haggard, ‘Mr. Rider Haggard’s Farm Diary’, *Lowestoft Journal*, 3 June 1899, 2.

27

Amanda Claybaugh , The Novel of Purpose: Literature and Social Reform in the Anglo-American World (Ithaca: Cornell University Press, 2007), 34 .

28 Although Haggard had included fictional critiques of the Gladstone government in previous novels, he had never so directly tried to intervene in a political debate. See Katz, *op. cit.* (note 12), 50.

29 Charles Longman, ‘Letter to Rider Haggard’, 14 September 1898 (Rider Haggard Collection, Columbia University Libraries Manuscript Collections).

30 ‘Saturday Review of Books and Art’, *New York Times*, 14 January 1899, 13.

31 Quoted in Etherington, *Imperium, op. cit.* (note 13), 22.

32 Longman, *op. cit.* (note 29).

33 Haggard, *op. cit.* (note 1), 47.

34 MacLeod, *op. cit.* (note 3), 115.

35

Spalding Lewis , ‘Romancing the Zulu: H. Rider Haggard, “Nada the Lily”, and Salvage Ethnography’, English in Africa, 39, 2 (2012), 69–84 .

36 Longman, *op. cit.* (note 29).

37 H. Rider Haggard, ‘Dr. Therne (To the Editor of the Spectator)’, *The Spectator*, 10 December 1898, 15.

38 ‘Publications To-Day’, *The Times*, 20 October 1898, 9.

39

Ellen Miller Casey , ‘Weekly Reviews of Fiction: The “Athenaeum” vs. the “Spectator” and the “Saturday Review”’, Victorian Periodicals Review, 23, 1 (1990), 8–12: 10 .

40 ‘Novels of the Week’, *The Spectator*, 3 December 1898, 836.

41
*Ibid*.

42 Haggard, *op. cit.* (note 37).

43 ‘Books and Bookmen’, *Manchester Guardian*, 17 December 1898, 5.

44 ‘The Literary Week’, *Academy*, 17 December 1898, 463.

45 ‘Dr. Therne’, *Athenaeum*, 24 December 1898, 891. For more on the journal’s stature, see Leslie A. Marchand , The Athenaeum: A Mirror of Victorian Culture (New York: Octagon Books, 1971).

46 ‘Recent Novels’, *The Times*, 28 December 1898, 10.

47 ‘Dr. Therne’, *Morning Post*, 7 January 1899, 7.

48 For its early history, see Richard D. Altick , Punch: The Lively Youth of a British Institution, 1841–1851 (Columbus: Ohio State University Press, 1997).

49 ‘Mr. Punch’s Dramatised Novels’, *Punch, or the London Charivari*, 1 February 1899, 59–60.

50

George Bernard Shaw , ‘Preface on Doctors’ [1913], Pygmalion and Three Other Plays (New York: Barnes and Noble, 2004), 163–245: 172.

51 For more on *The Outlook’*s early history and imperial politics, see Scott A. Cohen , ‘Imperialism Tempered by Expediency: Conrad and *The Outlook*
’, Conradiana, 41, 1 (2009), 48–66 .

52 ‘Mr. Rider Haggard and the Conscientious Objector’, *The Outlook*, 31 December 1898, 700–1.

53 ‘Reviews and Notices of Books’, *The Lancet*, 152, 3929 (1898), 1640–2.

54 ‘A Vaccination Romance’, *The BMJ*, 2, 1979, (1898), 1692–4. This title echoes the common refrain used at the time for the novels of H.G. Wells, the ‘scientific romance’. For more on the term, see Brian Stableford , Scientific Romance in Britain 1890–1950 (London: Fourth Estate, 1985).

55
*Ibid.*, 1694.

56
*Ibid.*, 1693.

57
*Ibid.*, 1693.

58 ‘Reviews and Notices of Books’, *op. cit.* (note 53), 1641.

59 Porter and Porter, *op. cit.* (note 8), 249 (footnote 111).

60

Tabitha Sparks , The Doctor in the Victorian Novel: Family Practices (Burlington: Ashgate, 2009), 19 .

61 ‘Antivaccinationists at Gloucester’, *The BMJ*, 149, 3845 (1897), 1285–1286: 1111.

62 ‘Reviews and Notices of Books’, *op. cit.* (note 53), 1640.

63 ‘Our London Letter’, *Medical News*, 24 December 1898, 846.

64 See, for example, ‘The Society for Promoting Christian Knowledge Acts for Two Years as a Society for Promoting Falsehoods’, on 24 August 1898, which accuses the SPCK of intentionally spreading lies about the smallpox vaccine’s efficacy.

65 ‘Dr. Therne’, *The Star*, 9 December 1898, 1.

66
*Ibid.*

67 ‘Fairy Tales’, *The Star*, 2 November 1898. The page number for this article is unknown, as the article was pasted in a book of cuttings collected by Alfred Milnes, housed at the London School of Hygiene and Tropical Medicine.

68
*Ibid.*, 1.

69 Porter and Porter, *op. cit.* (note 8), 239.

70 ‘Dr. Therne’, *Vaccination Inquirer*, 2 January 1899, 11–12.

71 Charles Longman, ‘Letter to Rider Haggard’, 9 February 1899 (Rider Haggard Collection, Columbia University Libraries Manuscript Collections).

72 ‘S.P.C.K. Parcel’, *Oxford Times*, 1 September 1900, 10.

73

Linda K. Hughes and Michael Lund , The Victorian Serial (Charlottesville: University of Virginia Press, 1991).

74

Jane Platt , Subscribing to Faith? The Anglican Parish Magazine 1859–1929 (London: Palgrave Macmillan, 2015), 99 .

75 Quoted in *ibid.*, 102.

76

Aileen Fyfe , Science and Salvation: Evangelic Popular Science Publishing in Victorian Britain (Chicago and London: University of Chicago Press, 2004).

77

W.O.B. Allen and Edmund McClure , Two Hundred Years: The History of the Society for Promoting Christian Knowledge, 1698–1898 (London: Society for Promoting Christian Knowledge, 1898), 195 .

78
*Truth*, 25 January 1900, 181.

79

Graham Law , Serializing Fiction in the Victorian Press (London: Palgrave Macmillan, 2000), 134 .

80 ‘Literary Notices’, *Wakefield and West Riding Herald*, 27 January 1900, 5.

81 ‘A rat, a rat. Dead. For a ducket,’ *East London Advertiser*, 20 January 1900, 3.

82
*Truth, op. cit.* (note 78).

83
*Truth, op. cit.* (note 78).

84
*Truth, op. cit.* (note 78).

85 Quoted in Durbach, *op. cit.* (note 10), 45.

86 Quoted in Durbach, *op. cit.* (note 10), 45.

87 ‘Anti-Vaccinators Meet at Lincoln and Expose the Defects of the New Act’, *Morning Leader*, 8 November 1898, 5.

88
*Ibid.*, 5

89 ‘A Novel with a Purpose’, *The Lancet*, 159, 4109 (1902), 1548.

90
*Ibid.*

91
*Ibid.*

92 Quoted in H. Rider Haggard , Dr. Therne: Anti-Vaccinist (London: George Newnes, Limited, 1902), ix.

93 The 200 000 figure comes from D.E. Whatmore, *H. Rider Haggard: A Bibliography* (London: Mansell Publishing Limited, 1987), 34; Bridget Griffen-Foley , ‘From Tit-Bits to Big Brother: A Century of Audience Participation in the Media’, Media, Culture and Society, 26, 4 (2004), 533–48.

94 ‘List of New Books and Reprints’, *Times Literary Supplement*, 9 May 1902, 134; ‘George Newnes (Limited)’, *Practical Teacher*, 23, 2 (1902), 109.

95
*Vaccination Inquirer*, 1 November 1901, 135.

96 For example, see Alfred Russel Wallace’s *Vaccination a Delusion, Its Penal Enforcement a Crime* (London: Swan Sonnenschein and Co., 1898). As Martin Fichman and Jennifer E. Keelan have found, anti-vaccinationists were able to compose reasonably compelling evidence (to the standards of the time) using statistics to warn of vaccination’s dangers. See their ‘Resister’s Logic: The Anti-Vaccination Arguments of Alfred Russel Wallace and Their Role in the Debates over Compulsory Vaccination in England, 1870–1907’, *Studies in History and Philosophy of Biological and Biomedical Sciences*, 38, 3 (2007), 585–607.

97

Arthur Phelps , Lord Dunchester, or the End of Dr. Therne: An Autobiography (London: Swan Sonnenschein and Co., 1901), 76 .

98

Laurence Rothfield , Vital Signs: Medical Realism in Nineteenth-Century Fiction (Princeton: Princeton University Press, 1994), 148 .

99 Beck, *op. cit.* (note 9), 314.

100 Quoted in ‘The Shifty Claims of Vaccination: Important Proposal by Mr. G. Bernard Shaw. A Challenge to Examine the Statistics’, *Vaccination Inquirer,* 1 November 1901, 145–6. For a look at Shaw’s role during this outbreak, see Bernard F. Dukore , ‘Bernard Shaw and the Smallpox Epidemic of 1901–2’, SHAW: The Journal of Bernard Shaw Studies, 40, 2 (2020), 285–97.

101 Quoted in *ibid.*, 146.

102 Phelps, *op. cit.* (note 97), 31.

103 ‘The End of Dr. Therne’, *Vaccination Inquirer*, 2 December 1901, 151.

104

Beatrice E. Kidd and M. Edith Richards , Hadwen of Gloucester: Man, Medico, Martyr (London: John Murray, 1933), 163 .

105 Porter and Porter, *op. cit.* (note 8), 249 (footnote 111).

106 Arthur Phelps, letter to Harriet Crompton, 1 March 1900, in the possession of Penelope Forrest. I am indebted to Penelope Forrest (née Phelps) – a great-granddaughter of Arthur Phelps – for making these papers available to me. Additionally, my thanks go to Anthea Boylston, another Phelps great-granddaughter, who shared her time and resources with me.

107 See, for example, ‘The Psychology of Antivaccination’, The BMJ (1901) v.1(2105), p. 1119. https://www.ncbi.nlm.nih.gov/pmc/articles/PMC2400635/?page=1.

108 Phelps, *op. cit.* (note 97), 7.

109 MacLeod, *op. cit.* (note 3), 115 (footnote 29).

110 Phelps, *op. cit.* (note 106).

